# Lipid Metabolism Is Dysregulated before, during and after Pregnancy in a Mouse Model of Gestational Diabetes

**DOI:** 10.3390/ijms22147452

**Published:** 2021-07-12

**Authors:** Samuel Furse, Denise S. Fernandez-Twinn, Davide Chiarugi, Albert Koulman, Susan E. Ozanne

**Affiliations:** 1 University of Cambridge Metabolic Research Laboratories and MRC Metabolic Diseases Unit, Wellcome-MRC Institute of Metabolic Science, University of Cambridge, Addenbrooke’s Treatment Centre, Keith Day Road, Cambridge CB2 0QQ, UK; sf615@cam.ac.uk (S.F.); df220@cam.ac.uk (D.S.F.-T.); 2 Core Metabolomics and Lipidomics Laboratory, Wellcome-MRC Institute of Metabolic Science, University of Cambridge, Addenbrooke’s Treatment Centre, Keith Day Road, Cambridge CB2 0QQ, UK; 3 Biological Chemistry Group, Jodrell Laboratory, Royal Botanic Gardens Kew, London TW9 3AD, UK; 4 Bioinformatics and Biostatistics Core, Wellcome Trust-MRC Institute of Metabolic Science, University of Cambridge, Addenbrooke’s Treatment Centre, Keith Day Road, Cambridge CB2 0QQ, UK; dc702@medschl.cam.ac.uk

**Keywords:** gestational diabetes, lipid metabolism, lipid traffic analysis, lipidomics, hyperglycaemia, mouse model

## Abstract

The aim of the current study was to test the hypothesis that maternal lipid metabolism was modulated during normal pregnancy and that these modulations are altered in gestational diabetes mellitus (GDM). We tested this hypothesis using an established mouse model of diet-induced obesity with pregnancy-associated loss of glucose tolerance and a novel lipid analysis tool, Lipid Traffic Analysis, that uses the temporal distribution of lipids to identify differences in the control of lipid metabolism through a time course. Our results suggest that the start of pregnancy is associated with several changes in lipid metabolism, including fewer variables associated with de novo lipogenesis and fewer PUFA-containing lipids in the circulation. Several of the changes in lipid metabolism in healthy pregnancies were less apparent or occurred later in dams who developed GDM. Some changes in maternal lipid metabolism in the obese-GDM group were so late as to only occur as the control dams’ systems began to switch back towards the non-pregnant state. These results demonstrate that lipid metabolism is modulated in healthy pregnancy and the timing of these changes is altered in GDM pregnancies. These findings raise important questions about how lipid metabolism contributes to changes in metabolism during healthy pregnancies. Furthermore, as alterations in the lipidome are present before the loss of glucose tolerance, they could contribute to the development of GDM mechanistically.

## 1. Introduction

Gestational diabetes (GDM) remains the most common cause of complications in human pregnancy [1]. The biggest risk factor for GDM is obesity, with approximately 30% of women with a BMI > 30 at the start of pregnancy developing GDM [2,3,4] compared to around 8% across the general UK population [1]. There is also accumulating evidence that maternal obesity-driven GDM leads to long-term (programmed) effects in the offspring [5,6,7,8,9,10,11], suggesting that GDM and its risk factors have inter-generational effects. There is an increased risk of both cardio-vascular disease and type 2 diabetes mellitus (T2DM) in the decade after delivery in individuals who developed GDM [12,13,14,15,16]. Research in this area includes a systematic review of over 5 million women across nine studies that found that women who developed GDM had a two-fold increased risk of cardiovascular events independent of developing T2DM [17].

The dangerous effects on mother and baby when GDM is poorly controlled and the increasing prevalence of the condition have led to investigation of its molecular origins. The hyperglycaemia and hyperinsulinaemia associated with GDM have received much attention, both for predictive models [18,19,20] and diagnosis [21,22], and have been shown to be associated with a number of genetic risk factors [23]. However, the hyperglycaemia associated with GDM is not screened for until the second trimester in humans [24]. Thus, although it remains the principal diagnostic indicator of GDM, hyperglycaemia is likely to be the effect and not the molecular origins of the condition. Several groups have shown that T2DM is accompanied by changes in lipid metabolism that can precede changes in glycaemic dysregulation, some of which have been suggested as having a causal relationship with T2DM [25,26], leading to questions about the role of lipid metabolism in GDM. Recent reports have shown that both the lipoprotein and the lipid composition of blood serum are altered in humans who go on to develop GDM, at least 10 weeks before formal diagnosis of the condition [27,28,29]. Notably, the hyperglycaemia associated with GDM can occur either or both pre- and post-prandially, and the prevalence of the condition is not evenly spread across ethnic groups and is higher in obese women, suggesting that GDM as currently described has several related but different origins.

**Figure 1:**
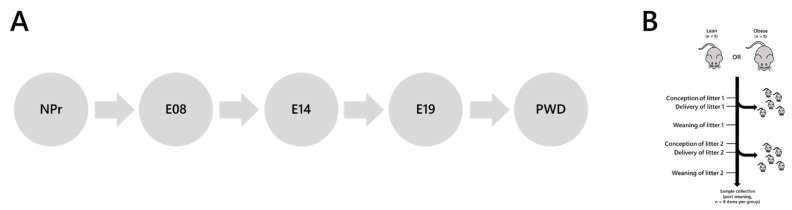
Mouse model and lipid collection points used in the present study. (**Panel A**) Collection time points used in the present study. (**Panel B**) Schematic representation of the mouse model used in this study [5,6,7]. NPr, not pregnant; E08, 8th day of pregnancy; E14, 14th day of pregnancy; E19, 19th day of pregnancy; PWD, post-weaning dam.

In the current study, we used a mouse model of diet-induced obesity that develops impaired glucose tolerance during pregnancy to test the hypothesis that the normal pregnancy-associated changes in lipid metabolism are modulated during a GDM pregnancy. We further hypothesised that the changes in lipid metabolism would be present before hyperglycaemia developed. We therefore determined the lipid composition (lipidome) of serum from five time points before, during and after pregnancy using high-resolution direct infusion mass spectrometry (DI-MS) (for schematic, see Figure 1). Fatty acid analysis was used to test for differences driven by diet, and applying our novel traffic analysis [30,31] allowed us to plot the temporal distribution of molecular species longitudinally.

## 2. Results

### 2.1. Metabolic Phenotyping

**Figure 2:**
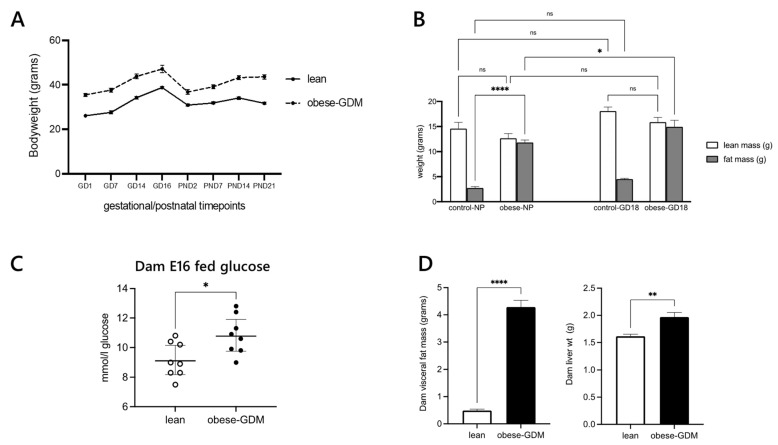
Dam morphometric and metabolic status data. (**Panel A**) Body weight trajectory from pre-gestation to weaning; (**B**) a comparison of body composition prior to mating (NP) with E18 gestation; (**C**) fed blood glucose at day 18 of gestation; (**D**) maternal total dissected visceral fat weights and liver weights at the end of the study (postnatal day 21). Data are presented as ±SEM, *n* = 8 per group. Significant differences are denoted **** *p* < 0.001, ** *p* < 0.01, * *p* < 0.05. *ns*, not significant, i.e., *p* > 0.05.

The obese dams were both heavier and fatter than control dams before pregnancy, in late gestation and through lactation till weaning (Figure 2A,B). Hyperglycaemia was present in our mouse model of obese GDM at E16, roughly equivalent to the point at which humans are tested for hyperglycaemia in pregnancy (~24–28w) (Figure 2C). Obese dams also had increased adipose tissue and liver weights at weaning (Figure 2D). There was no difference in litter size at birth, but pups born to obese dams were significantly smaller than controls (Table 1). 

Measures of lipoproteins and hormones in the circulation provided the first direct suggestion that lipid metabolism was altered at the same times as the hyperglycaemia at E16 (Table 2). They indicated that triglycerides (TGs) were less abundant and cholesterol was more abundant in the circulation of mice with GDM. The obese dams were also hyperinsulinaemic and hyperleptinaemic. The reduced abundance of TGs in the circulation of the obese-GDM group was surprising based on their dietary intake. The intake of fat is considerably higher in the obese-GDM (experimental) group; lean (control) mice ate around 5.7 g of chow diet per day (2.56 kcal/g; fat, 7.42% (kcal); RM1, Special Diets Services, Witham, UK), whereas obese mice ate around 5.5 g of the HF pellet (6.79 kcal/g; fat, 45% (kcal); 45% AFE Fat, Special Diets Services, Witham, UK) as well as 3 g of condensed milk per day (8% fat [*w*/*w*]; Nestle, fortified with mineral and vitamin mix AIN93G), with the latter presenting a higher overall fat intake than the chow diet by around an order of magnitude. The discrepancy between TG abundance in the circulation compared to dietary intake suggested that the control of lipid/fatty acid (FA) distribution and metabolism differed between the two groups of animals. We therefore investigated whether the abundance of FAs in the circulation was driven entirely by the dietary supply to the two groups.

### 2.2. Fatty Acid Regulation

**Figure 3:**
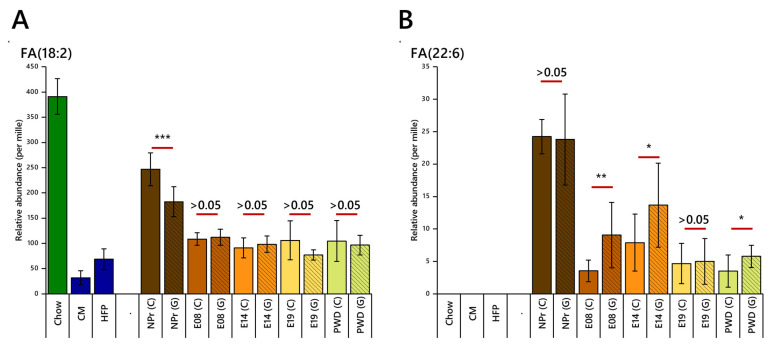

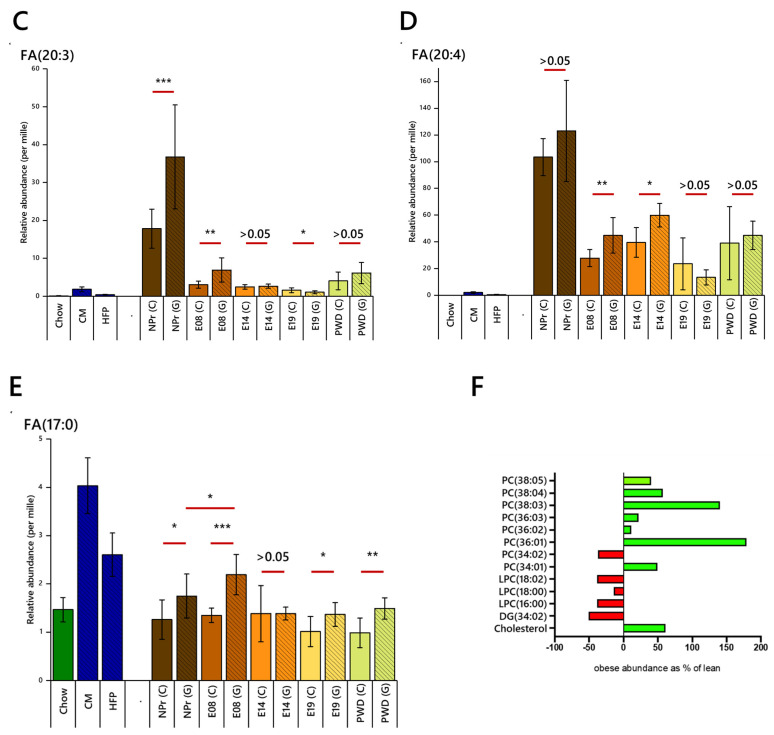
FA abundance (per mille) for fatty acids in the dietary intake and at non-pregnant (NPr), 8, 14 and 19 days of gestation, and post-weaning presented as means ± SD. (**Panel A**) Linoleic acid, FA(18:2); (**B**) docosahexaenoic acid (DHA), FA(22:6); (**C**) linolenic acid, FA(20:3); (**D**) arachidonic acid FA(20:4); (**E**) margaric acid, FA(17:0); (**F**) abundance of dietary-intake-associated variables between control and obese-GDM groups before pregnancy. PC, phosphatidylcholine; PE, phosphatidylethanolamine; LPC, *lyso*-phosphatidylcholine. *** *p* < 0.001, ** *p* < 0.01, * *p* < 0.05. NPr, not pregnant; E08, 8th day of pregnancy; E14, 14th day of pregnancy; E19, 19th day of pregnancy; PWD, post-weaning dam.

Linoleic acid, an essential fatty acid with configuration FA(18:2), was one of the most abundant fatty acids in the circulation before pregnancy (20–25%) and fell considerably by E08 in both groups to (~12%, Figure 3A). This happened despite the dietary supply of that FA remaining unchanged. Furthermore, although the proportion of linoleic acid in the chow diet was considerably higher than the HFD, the higher overall intake by the obese-GDM group meant that the dietary supply of FA(18:2) was similar. These results suggest that the abundance of this FA in the circulation is controlled very similarly in both groups in the pre- and post-conception periods, but that the early pregnancy period in particular requires considerable re-organisation of the distribution of FAs such as FA(18:2), which is more important than the supply of the FA.

The relative abundance of docosahexaenoic acid (DHA), FA(22:6), also fell at the start of pregnancy but remained generally higher in the obese-GDM group throughout pregnancy. However, at E19, the abundances of DHA were similar for the two groups (Figure 3B). These patterns suggested that the control over the abundance of this FA is different between groups and change differently through pregnancy.

Polyunsaturated fatty acid dihomo-γ-linolenic acid, FA(20:3), was also more abundant in the obese-GDM group before pregnancy, with this difference being maintained until E08 despite a considerable reduction in abundance in both groups at the start of pregnancy (Figure 3C). Interestingly, the abundance of this FA fell further by E19 in the obese-GDM group, such that the lean group had a higher abundance than the obese-GDM group by the end of pregnancy thus inverting the pattern from non-pregnant and early pregnancy. Arachidonic acid, FA(20:4), represents 10–15% of the FA in the circulation of non-pregnant mice despite a low abundance in both diets, and was not different between the groups (Figure 3D). However, a difference between the groups emerged during pregnancy, with it being more abundant in the obese-GDM group until E14. Notably, the abundance of both FA(20:3) and FA(20:4) in the circulation was considerably higher than the dietary supply. The combination of a higher proportion of these two fatty acids in the high-fat obesogenic diet and the higher overall fat intake by the obese-GDM group meant that the daily dietary supply of these FAs is at least 10× higher in the obese-GDM group. Despite this, FA(20:3) was only around twice as abundant in the circulation of the obese-GDM group, and FA(20:4) was at most 30% higher in the GDM (E14), with several time points showing no difference between groups.

The serum concentrations of margaric acid, FA(17:0), remained fairly constant within each group during the period studied. However, FA(17:0) was ~30% higher in the obese-GDM group before conception, at E08 and at weaning (Figure 3E). Thus, the same pattern of abundance is maintained at these points despite a 50× greater dietary supply for the obese-GDM group. Furthermore, the relative abundance of margaric acid increased (*p* < 0.05) after conception, but only in the obese-GDM group. This suggests that there may be low-level changes in the abundance of this FA, but more importantly that the two phenotypes differ in this regard. There is no appreciable difference between groups at E14, showing that despite both a difference in dietary supply of this FA and different patterns through other parts of pregnancy, the two groups maintained good control over the abundance of this FA, such that its abundance in the circulation remains the same or is only marginally different (Figure 3E).

These results show both similarities and counter-intuitive differences in the abundance of FAs between groups and before, during and after pregnancy. These patterns emerge despite large differences in dietary supply between groups. These effects were observed across a range of FAs; saturated and unsaturated, odd and even, dietary and de novo. This showed that the control of lipid metabolism differs between the groups in a way not driven purely by current dietary intake. The higher abundance of FA(20:3) and FA(20:4) in the obese-GDM group is reflected in the higher abundance of PUFA-containing PCs before conception (Figure 3F), suggesting that the temporal control of lipid metabolism is different between the two groups as well as the FA abundance *in circulo*. This led us to the hypothesis that the control of lipid metabolism changed during pregnancy and that this was also changed by GDM before, during and after gestation. We tested this hypothesis using a lipid class analysis followed by an analysis of lipid traffic (LTA).

### 2.3. Lipid Abundances by Head Group/Class

**Figure 4:**
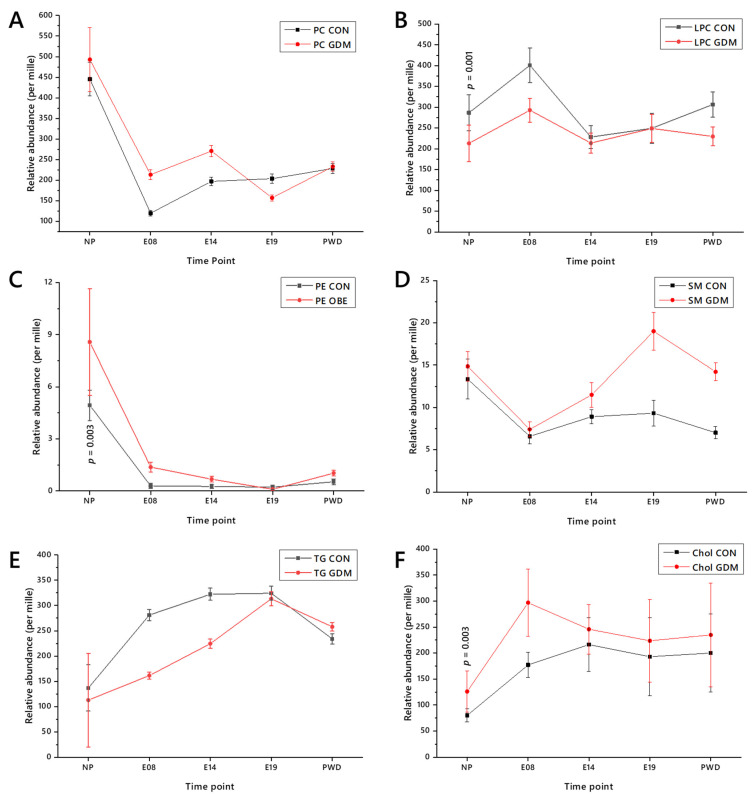
The abundance of lipid classes in the circulation through pregnancy presented as means ± SD. (**Panel A**) Phosphatidylcholine; (**B**) *lyso*-phosphatidylcholine; (**C**) phosphatidylethanolamine; (**D**) sphingomyelin; (**E**) triglyceride; (**F**) Cholesterol. E08. 8th day of pregnancy; E14, 14th day of pregnancy; E19, 19th day of pregnancy; NP, not pregnant; PWD, post-weaning dam.

The relative abundance of major lipid classes changes considerably through both normal pregnancy and GDM (Figure 4). Structural lipids such as phosphatidylcholine (PC), phosphatidylethanolamine (PE) and sphingomyelin (SM) decreased after conception, whereas triglycerides (TGs) and cholesterol (Chol) increased in relative abundance. Conversely, *lyso*-phosphatidylcholines (LPC) rose. In general, the patterns of change in relative abundance of the two groups are similar, notwithstanding the fact that the overall abundance was different—for example, the abundance of TG increases at the start of pregnancy but increases much more in the lean group than in the obese one. Exceptions include E19, in which the abundance of PC in the circulation of the lean group is greater that of the obese one (Figure 4A). These changes in abundance support the hypothesis that lipid metabolism differs between both pregnant and non-pregnant states and between control and GDM groups.

Lipid class abundance provides a useful insight into lipid metabolism but is limited, as each class comprises a number of isoforms. Analysis at the isoform level not only affords better resolution, but patterns within sub-classes may also be observed and investigated. We therefore further tested the hypothesis that lipid metabolism is changed in the pregnant versus non-pregnant state as well as between control and GDM groups, by analysing lipids through the whole time course using our novel Lipid Traffic Analysis (LTA), which we have recently updated [31].

### 2.4. Lipid Traffic Analysis

**Figure 5:**
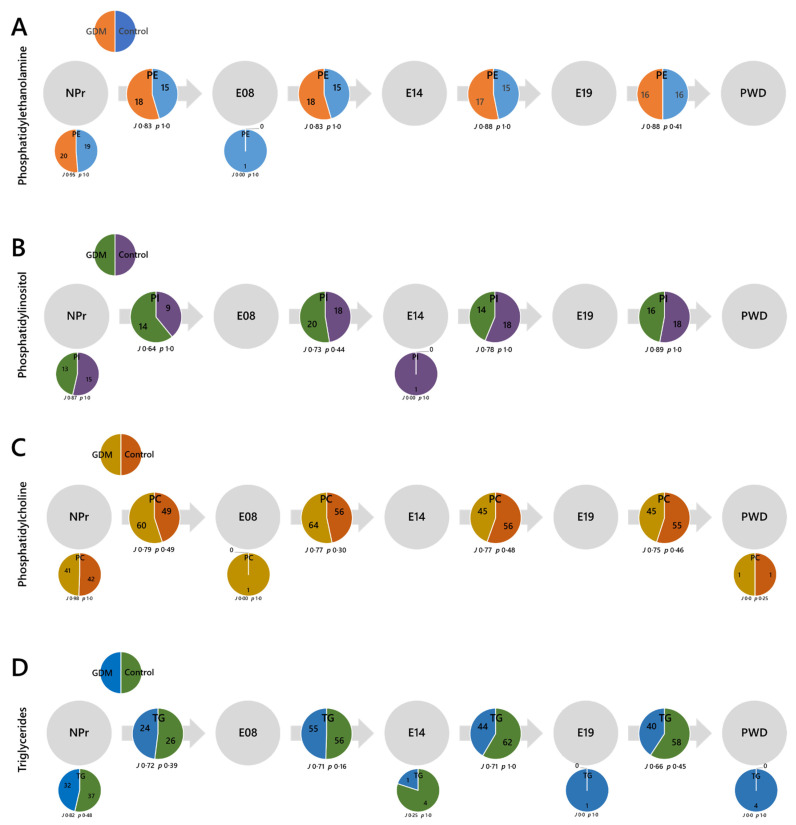
Switch analyses of phosphatidylethanolamine (PE), phosphatidylinositol (PI), phosphatidylcholine (PC) and triglyceride (TG) classes from before conception to after weaning. (**Panel A**) PE; (**B**) PI; (**C**) PC; (**D**) TG. ***U***-type variables are shown in small pie charts under the appropriate time point, where found. ***B***-type lipids are shown in pie charts on the arrow between the appropriate time points. ***A***-type variables: TGs (22:18, *J* 0·82, *p* 1·0); PEs (14:16, *J* 0·88, *p* 1·0); PCs (45:40, *J* 0·81, *p* 0·49); PIs (9:10, *J* 0·90, *p* 1·0). NPr, not pregnant; E08, 8th day of pregnancy; E14, 14th day of pregnancy; E19, 19th day of pregnancy; PWD, post-weaning dam.

The switch analysis part of the LTA (Figure 5) revealed considerable changes in lipid metabolism after conception. There were considerable numbers of ***U***-type variables in the NPr timepoint (small pie chart), which represent lipid isoforms that are present pre-conception but decrease at the start of pregnancy (Figure 5A–C). Notably, despite an increase in the relative abundance of TG after conception (Figure 4E), the number of variables decreases (Figure 5D). PE, PI and TG all show decreases in palmitate-containing isoforms and PE, PI and PC all show decreases in the number of odd-chain FA-containing (OCFA) isoforms after conception. The isoforms 32:0 and 32:1 of PE and PI disappeared at the start of pregnancy, as did TGs 48:1, 48:2, 50:2 and 50:3. FA(16:0) isoforms of TG such as these are associated with de novo lipogenesis (DNL) [32], and so these, along with PE and PI variables containing the same FAs, indicate that DNL falls at the start of pregnancy.

**Figure 6:**
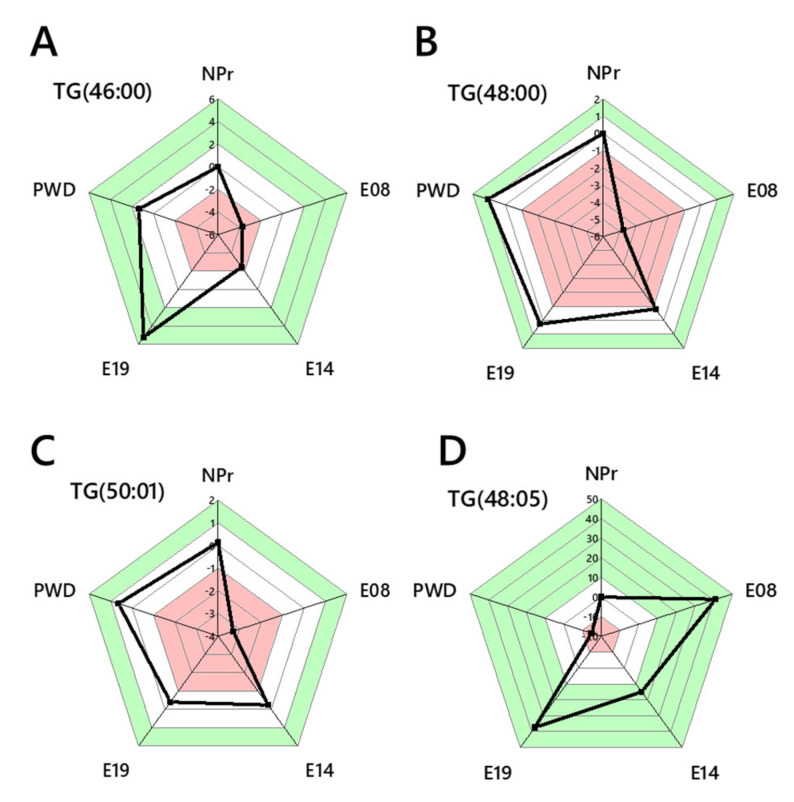
Abundance analysis of TG variables measured at all time points. (**Panel A**) Abundance analysis of TG(46:0); (**B**) TG(48:0); (**C**) TG(50:1); (**D**) TG(48:5). Panels A–C represent TGs associated with *de novo* lipogenesis, whereas D represents those of mixed and/or dietary origin. NPr, not pregnant; E08, 8th day of pregnancy; E14, 14th day of pregnancy; E19, 19th day of pregnancy; PWD, post-weaning dam.

The reduction in palmitate-containing isoforms of PE, PI and TG in the circulation at the start of pregnancy is also counter-intuitive given the evidence that the relative abundance of TGs increases by E08 in both groups, more so in the lean group. We therefore tested whether this was also the case in TGs that are established as markers of DNL but that were measurable throughout pregnancy. TG(46:0, 48:0, 50:1) decreased in abundance significantly more in the obese-GDM group than the controls by E08, before increasing steadily and returning to around the same abundance as the lean group by weaning (Figure 6A–C). The effect of a lower abundance of DNL/palmitate-containing TGs in the obese-GDM group is not observed with non-DNL isoforms (e.g., TG(48:5), Figure 6D), suggesting that DNL in particular is altered at the start of pregnancy, changing the supply of palmitate for onward lipid metabolism. Importantly, palmitate-containing isoforms of PC were maintained during pregnancy. PC(30:0, 32:0, 34:1) were present throughout pregnancy and PC(34:0) was found again from E14 onwards.

The suggestion that DNL is reduced, as indicated by changes in at least two major phospholipid classes and TGs, but not PCs, suggests that both fatty acid and lipid metabolism are modulated early in pregnancy. The error-normalised fold change (ENFC) between control and obese-GDM groups for non-DNL TGs, e.g., TG(48:5), showed a different pattern to palmitate-containing species, but is consistent with the general observation that FA metabolism has been reorganised. The results from the switch analysis were used to investigate this further by plotting the spatial distribution of lipid variables in both phenotypes.

The switch analysis (Figure 5A–D) showed that different numbers of ***B***-type variables are present in the two groups at the start of pregnancy, with more present in the obese-GDM group for PE, PI and PC. As there were roughly the same number of ***U***-type lipids at NPr, this showed that the temporal distribution of these head groups in the circulation was shortened in the control group, i.e., switched off earlier. The difference in ***B***-type variables between groups continues through pregnancy, with a decrease in the number of PE variables and an inversion for PI and PC, with many more ***B***-type PCs present in the control group in the late pregnant and weaning stages. There were marginally more ***B***-type TGs in the control group at the outset of pregnancy, but nearly 50% more by weaning. Thus, in normal pregnancy, there is a considerable reorganisation of the PC, PI and TG profile in the circulation, with a scaling back of the number of variables early in pregnancy, some of which return to pre-pregnancy levels by E14. Importantly, PC 33:3, 33:5, 36:5, 42:6, and 44:7 were found in the circulation of the obese-GDM group up to E14, after which point they were not detectable, whereas the same isoforms were found in the control group only from E14. The same pattern was observed for PI(40:6) and TG 38:0, 39:1, and 56:7. The number of TGs fell from around E14 in the obese-GDM group, whereas in the control group, it increased. Taken together, this shows that the obese-GDM systems reduced the abundance of a variety of both TGs and phospholipids around the time that the healthy pregnancies increased them.

## 3. Discussion

This study addressed the hypothesis that lipid metabolism changes during normal pregnancy and that the control of these changes would differ in those affected by GDM. We demonstrate substantial changes in lipid metabolism during healthy and GDM pregnancy, even earlier than changes in glucose. However, unlike well-studied changes in carbohydrate metabolism [33,34,35], changes in lipid metabolism remained after delivery in GDM pregnancy. Our longitudinal analysis also demonstrates that the control of lipid metabolism also differs during a GDM pregnancy, providing evidence to support long-standing suggestions that dysregulation of lipid metabolism could lead to the development of GDM and T2DM [25,36]. Furthermore, these novel findings highlight that differences are present before hyperglycaemia develops in both humans and mice [28,29]. An advantage of the longitudinal structure of the present analysis is that it has been able to identify changes to the timing of changes in lipid metabolism. This includes evidence for changes to processes such as DNL.

The evidence for a reduction in DNL at the start of pregnancy is important as it represents a significant change in the supply of energy available from the maternal circulation. This may also indicate that glucose is being oxidised instead, perhaps by the developing fetus, rather than turned into FAs endogenously. Greater oxidation of glucose towards the end of pregnancy has been reported previously [33]. The evidence for these changes to DNL occurring before the known development of pregnancy-associated insulin resistance is accompanied by different timing in the control of GDM systems. Higher abundance of markers of DNL was found in the obese-GDM group in the present study and in human studies of lipid metabolism before GDM [29]. This shows that a repressed modulation of DNL is associated with GDM. Furthermore, as the improvements in clinical outcomes of GDM treated through lifestyle changes are consistent with improved lipid metabolism, this evidence offers a range of possible targets for therapeutic intervention, including more specific changes to diet and lifestyle during and after pregnancy.

Both diets were predominantly composed of carbohydrates, so DNL continued in the livers of both groups throughout pregnancy. It is unclear how this is restructured in the obese-GDM animals, however the changes made appear to be insufficient, as the abundance and number of DNL-associated variables were much higher in the obese-GDM system than in the control (healthy) one. One likely explanation is that oxidative rates of glycolysis are lower in obese GDM, thus leaving more substrates for DNL. Another possibility is that the excess DNL FAs are not trafficked to the adipose as quickly as in the control group. Adipose increases in volume considerably in obese-GDM, with this study finding clear evidence of weight gain during pregnancy in the obese-GDM group, and thus the role of adipose tissue in lean and obese pregnancy is important for further understanding of triglyceride metabolism in pregnancy and GDM.

The observations made about OCFA-containing lipid species in the present study are less easy to interpret than for DNL variables, although they show a similar pattern overall. As with saturated and mono-unsaturated isoforms of TG and the polyunsaturated isoforms of PC and PI, several OCFA-containing PCs are not switched off in early pregnancy in the obese-GDM group but are in the control group. These same variables are reduced in the obese-GDM group just as they are increased again in the control group, around E14. Dietary intake represents one source of OCFAs; however, it can also be produced endogenously through the activity of *Hacl1* [37,38].

Importantly, the reduction in the variety of OCFA-containing lipid isoforms in both groups at the start of pregnancy (albeit less effectively in the obese-GDM group) occurs without a large change in the overall abundance of FA(17:0). The evidence that the relative abundance of this FA remains consistent in either group is consistent with the DNL markers discussed above. This further suggests that the FA profile is remodelled considerably as a normal part of healthy pregnancy and that this too is dysregulated under GDM. Changes to the abundance of species with OCFAs is of interest in the light of evidence that these are associated with lower risk of cardio-metabolic disease [39] in non-pregnancy. However, this relationship may be inverted in pregnancy, with the higher abundance in the circulation of at least one OCFA-containing species associated with the development of GDM later in pregnancy [29]. The reduction in the variety in OCFA-containing species in the circulation around the beginning of normal pregnancy is consistent with this, as is the weaker scaling-back of OCFA-containing species in GDM.

Although several palmitate-containing PC variables remain in the circulation after conception, unlike the same isoforms of PE and PI, PUFA-containing PCs were altered both in pregnancy and between groups. Several PUFA-PCs remain in the circulation of the obese-GDM group for longer after conception than they do in the control group and are reintroduced into the circulation just before E14, around the point at which they finally disappear from the GDM animals. This shows a difference in control in the two systems, with both a normal change in pregnancy and a dysregulation in obese-GDM. These changes may have a structural effect through the role of PCs in the fluidity of membranes [40,41]. The role of PC as a storage and transport vehicle for PUFAs [42] may also be important in both the timing of changes to lipid metabolism and supply of nutrients to the fetus. LPC is produced from PC via enzyme-driven hydrolysis and has been found to have a potential role in delivering long-chain polyunsaturated fatty acids (LCPUFAs) to the fetal circulation [43]. PUFAs are essential for supporting fetal growth and in particular, LCPUFAs support visual and cognitive development [44]. So, while PC was more abundant in the obese-GDM circulation compared to the lean, this did not translate to an increase in LPC, in fact it was reduced. LCPUFAs, LPC and *lyso*-phosphatidylethanolamine (LPE) are selectively transported from maternal circulation into cord blood and have been shown to be positively correlated with birth weight [45]. Therefore, the significant reduction in LPCs at the start of the GDM pregnancy observed in this study could be responsible for the fetal growth restriction observed in this mouse model [46].

A limitation of the current study is that it is focused on serum; this is the only tissue that could be sampled longitudinally from the same animal. This means the lipids measured represent the average of the circulation, limiting the scope for characterising the contribution of tissues that dominate lipid metabolism, such as the liver and adipose. Studies that use tissues that cover the bulk of lipid metabolism, throughout the organism, are mounting but focus on a single time point. They show that lipid metabolism within individuals differs between feeding types and between paternal programming groups [30,31]. Thus, further work in this area could include a system-wide analysis of lipid metabolism at time points in pregnancy.

An understanding of the relationship between maternal metabolism and/or metabolic phenotype and short- and long-term outcome for the infants is important. Extensive evidence in both humans and animal models has shown an association between obesity during pregnancy and offspring cardio-metabolic health. Furthermore, a growing number of studies show that obesity, including during pregnancy, is associated with dysregulated lipid metabolism [28,47]. However, more work is required (a) to define the causal pathways linking maternal lipid metabolism and offspring cardiometabolic health and (b) to establish the time windows and opportunities to reverse/correct them. The current studies identify potential lipid species and pathways that could be the focus of future studies to address these knowledge gaps.

## 4. Materials and Methods

### 4.1. Animal Model

All procedures were conducted in accordance with the UK Home Office Animal (Scientific Procedures) Act 1986 and the Animal Welfare & Ethical Review Body of the University of Cambridge and approved under project licence P5FDF0206, 23 March 2017. Animals were maintained at the university’s biomedical research facility as described previously [6,7,48]. Briefly, female C57BL/6 mice were fed either a control (RM1) or an obesogenic high-fat-high sugar diet from weaning (both diets manufactured by Special Dietary Services Ltd.; Witham, UK). Diet compositions have been described [10]. All dams experienced a first pregnancy to demonstrate proven breeder status. Maternal blood samples for the current study were collected longitudinally for the current study immediately prior to mating for the second pregnancy and then at E08, E14, E19 during pregnancy and then three weeks after birth (see Figure 1B); *n* = 8 dams per group were used. Dams’ body compositions just prior to mating for the second pregnancy and at E18 of gestation were measured by TD-NMR (Bruker, Germany).

### 4.2. Blood Glucose Measurement

On day 16 of pregnancy, blood was drawn from the tail for glucose measurements (AlphaTRAK2, Zoetis, Surrey, UK).

### 4.3. Lipidomics

#### 4.3.1. Isolation of the Organically Soluble Fraction for MS

Lipids, triglycerides and sterols were extracted together using a high-throughput platform described recently [48,49]. Briefly, aliquots of serum lithium heparin (20 µL) were placed along with blank and QC samples in the wells of a glass-coated 2.4 mL/well 96w plate (Plate+™, Esslab, Hadleigh, UK). Methanol (150 μL, HPLC grade, spiked with Internal Standards, see Table S1), was added to each of the wells, followed by water (500 µL) and DMT (dichloromethane, methanol (3:1) and triethylammonium chloride 500 mg/L; 500 µL). The mixture was agitated (96 channel pipette) before being centrifuged (3.2k× *g*, 2 min). A portion of the organic solution (20 µL) was transferred to a high-throughput plate (384w, glass-coated, Esslab Plate+™) before being dried (N_2 (g)_). The dried films were redissolved (TBME, 30 µL/well) and diluted with a stock mixture of alcohols and ammonium acetate (100 µL/well; propan-2-ol: methanol, 2:1; CH_3_COO·NH_4_ 7.5 mM). The analytical plate was heat-sealed and run immediately.

#### 4.3.2. Mass Spectrometry

Samples were infused into an Exactive Orbitrap (Thermo, Hemel Hampstead, UK), using a Triversa Nanomate (Advion, Ithaca, NY, USA). Samples were ionised at 1.2 kV in the positive ion mode. The Exactive started acquiring data 20 s after sample aspiration began. After 72 s of acquisition in positive mode, the Nanomate and the Exactive switched over to negative mode, decreasing the voltage to −1.5 kV. The spray was maintained for another 66 s, after which the analysis was stopped and the tip discarded, before the analysis of the next sample. The sample plate was kept at 15 °C throughout the acquisition. Samples were run in row order.

#### 4.3.3. Data Processing

Raw high-resolution mass-spectrometry data were processed using XCMS (www.bioconductor.org, accessed on 2 July 2021) and Peakpicker v2.0 (an in-house R script [50]). Lists of known species (by *m*/*z*) for both positive ion and negative ion mode were used (~8.5k species). Variables whose mass deviated by more than 9 ppm from the expected value, had a signal/noise ratio of <3, or had signals for fewer than 50% of samples were discarded. The correlation of signal intensity to concentration of human placenta, mouse liver, human serum and pooled human seminal plasma samples as QCs (0.25, 0.5, 1.0×) were used to identify the lipid signals, the strength of which was linearly proportional to abundance (threshold for acceptance was a correlation of 0.75). Signals were then divided by the sum of signals for that sample and expressed per mille (‰). Zero values were interpreted as not measured. All statistical calculations were done on these finalised values. DIMS identified up to 492 lipid variables in positive ionisation mode and up to 607 lipid variables in negative ionisation mode in serum samples. The combination of these ionisation modes enabled us to verify the abundance of lipid classes that can be isobaric. PC and PE isoforms can be isobaric in positive ionisation mode, but are separable in negative ionisation mode, enabling verification of identification.

#### 4.3.4. Lipid Traffic Analysis

Lipid Traffic Analysis [30] v2.3 was used [31]. This consisted of identifying lipids as ***U***-type (unique to one compartment but found in one or both phenotypes), ***A***-type (found in all compartments in either phenotype) and ***B***-type (found in two metabolically adjacent compartments, such as Liver and Serum). The code for the switch analysis included alignment of lists and automated calculation of *J* and *p* values from binary lists and improved categorisation of lipid variables (including assessment of all TG-derived glycerides). The configuration of the ***U***-lipid, ***A***-lipid and ***B***-lipid sections of the code made running any of the three individual parts of the code feasible. Novel code was written in R(v3.6.x) and processed in RStudio(v1.2.5x). The full code for Lipid Traffic Analysis v2.3 can be found in the *Supplementary Information File S1*.

The analysis of the present study was similar to previous studies [30,31]. The tissues used were mapped to the known biological/metabolic network (Figure 1A). Categories for the switch analysis were ***A***, ***B*** and ***U*** lipid types. Variables were regarded as present if they had a signal strength >0 in ≥66% of samples per group.

### 4.4. Statistical Methods

Univariate and bivariate statistical analyses were calculated in Microsoft Excel 2016. Error-normalised fold change (ENFC) [30] was calculated in the LTA [31]. Graphs were prepared in Excel 2016 or OriginLab 2018. Calculations of Jaccard-Tanimoto Coefficients (JTCs, *J*) and associated *p*-values [30] were used as a non-parametric measure of the distinctions between lipid variables associated with phenotype(s). The *p*-value associated with each *J* represents the probability that the difference between the lists of variables for the two phenotypes occurred by random chance, representing both the number of variables only found in either of the two groups and the order of the binary list.

## 5. Conclusions

We conclude that lipid metabolism changes during healthy pregnancy to support adequate nutrient supply to the developing fetus. In particular, TG and cholesterol supply increases after conception and PCs and PEs (known substrates for the production of LPCs and LPEs) decline. As LPCs and LCPUFAs are essential for fetal growth and are positively linked to birth weight, their suppression in the circulation of mouse GDM pregnancies could explain the accompanying fetal growth restriction. The results presented here have important implications for our understanding of the control of metabolism during pregnancy and indicate a variety of potential therapeutic targets for lifestyle and pharmacological interventions to treat GDM and its consequences for the developing fetus. Finally, this study highlights the importance of correct control of lipid metabolism in pregnancy and supports the suggestion that GDM is driven by dysregulation of lipid metabolism that begins in early pregnancy, before the dysregulation of carbohydrate metabolism.

## Figures and Tables

**Table 1:** The effect of maternal obesity on litter size and pup weights at postnatal day 2. Data are expressed as mean ± SEM for litter size and medians and interquartile ranges for pup weights. Litter size was analyzed by 2-tailed unpaired t-test while pup weights were analyzed by Kolmogorov–Smirnov test and the significant effects of obesity are indicated; ** *p* < 0.01.

PND2	Control	Obese
Litter size	7.8 ± 0.5	5.5 ± 0.4 **
Pup weights (grams)	1.8 (1.65, 1.92)	1.45 (1.29, 1.6) **

**Table 2:** The effect of obesity on maternal serum metabolites at E18. Data are expressed as mean ± SEM. The effect of obesity by 2-tailed unpaired t-test and the significant effect of obesity on metabolites are indicated; * *p* < 0.05, ** *p* < 0.01.

E18	Control	Obese
FFA (μM)	831 ± 266	713 ± 125
TG (mM)	1.1 ± 0.1	0.7 ± 0.05 *
Cholesterol (mM)	1.3 ± 0.1	1.5 ± 0.1 *
Insulin (pM)	121.8 ± 34.8	278.4 ± 52.2 *
Leptin (pg/mL)	2171 ± 188	7778 ± 1390 **

## Data Availability

The MS dataset generated in the present study is available in Supplementary Information S5–11 of a previous study [49].

## Supplementary Materials

The following are available online at https://www.mdpi.com/article/10.3390/ijms22147452/s1.
